# Comparative Analysis of the Gut Microbiota of Thai Indigenous Chicken Fed House Crickets

**DOI:** 10.3390/ani15071070

**Published:** 2025-04-07

**Authors:** Nattanan Panjaworayan T-Thienprasert, Titiradsadakorn Jaithon, Pavit Klomkliew, Prangwalai Chanchaem, Thanathip Suwanasopee, Skorn Koonawootrittriron, Attawit Kovitvadhi, Pipatpong Chundang, Prapasiri Pongprayoon, Sutasinee Kityakarn, Patraporn Luksirikul, Sunchai Payungporn

**Affiliations:** 1 Department of Biochemistry, Faculty of Science, Kasetsart University, Bangkok 10900, Thailand; titiradsadakorn.j@ku.th; 2 Center of Excellence in Systems Microbiology, Department of Biochemistry, Faculty of Medicine, Chulalongkorn University, Bangkok 10330, Thailand; pavit.kw@gmail.com (P.K.); pc_redseed@hotmail.com (P.C.); 3 Department of Animal Science, Faculty of Agriculture, Kasetsart University, Bangkok 10900, Thailand; agrtts@ku.ac.th (T.S.); agrskk@ku.ac.th (S.K.); 4 Department of Physiology, Faculty of Veterinary Medicine, Kasetsart University, Bangkok 10900, Thailand; fvetawk@ku.ac.th (A.K.); pichandang@gmail.com (P.C.); 5 Department of Chemistry, Faculty of Science, Kasetsart University, Bangkok 10900, Thailand; fsciprpo@ku.ac.th (P.P.); fscistsn@ku.ac.th (S.K.); fscipplu@ku.ac.th (P.L.); 6 Center for Advanced Studies in Nanotechnology for Chemical, Food and Agricultural Industries, KU Institute for Advanced Studies, Kasetsart University, Bangkok 10900, Thailand

**Keywords:** gut microbiota, Thai indigenous chicken, house crickets, gut health, probiotics

## Abstract

The gut microbiota is pivotal to chickens’ overall health, influencing digestion, nutrient absorption, and immune function. Dietary compounds significantly impact gut microbiota composition. House crickets (*Acheta domesticus*) have emerged as an alternative protein source for animal feed, rich in proteins and beneficial fatty acids. This study compared the gut microbiota in the cecum and ileum of Thai indigenous chicken breeds (Betong Chicken, white feather with black bone chicken, and black feather with black bone chicken) fed with or without house crickets. Using Oxford Nanopore Technology of 16S rDNA, this study found a similar relative abundance of gut bacteria across groups, with dominant bacteria including Firmicute, Bacteroidetes, Proteobacteria, and Actinobacteria. LEfSe analysis identified differential abundance of beneficial bacteria, such as *Ruminococcaceae*, *Rikenella*, and Deferribacteres, in the cecum of the black feather with black bone chicken after cricket feeding. Additionally, *Lactobacillaceae* exhibited differential abundance in the ileum of this breed post-cricket diet. Consequently, this study provides new data into the gut microbiota of Thai indigenous chickens. It suggests that house cricket diets did not significantly alter microbiota diversity but may enhance beneficial bacteria in certain breeds.

## Simple Summary

This study investigated how a house-cricket-based diet affects the gut microbiota of different Thai chicken breeds. The results showed that while the cricket diet did not significantly change the overall diversity of gut bacteria, it increased beneficial bacteria in some breeds. This study provides new insights into the gut microbiota of Thai indigenous chickens and suggests that using house crickets in chicken feed could have positive effects on gut health without disrupting the balance of gut bacteria.

## 1. Introduction

The “microbiome” refers to the collection of microorganisms, including bacteria, viruses, fungi, and other microbes, that live in the same environment. These microorganisms can be found in various parts of the host such as the skin, mouth, and gut [1]. These microorganisms can have beneficial (symbiotic) or harmful (pathogenic) effects [2] on their host, and the complex interactions between the two are significant for the well-being of both humans and animals [3,4,5].

In poultry, the microbiome mainly exists within the gastrointestinal tract. It helps to break down feed, produce vitamins, and support the immune system. Therefore, the variety and composition of the gut microbiome are closely related to the health of poultry [6,7]. In chicken, the microbiome also plays a role in regulating the pH of the gut, preventing the overgrowth of pathogenic bacteria such as *Salmonella* and *Campylobacter* and involving bodily processes [8]. Therefore, disruption of the gut microbiome either using antibiotics or poor management practices such as nutrition malabsorption can lead to an overgrowth of harmful bacteria and increase the risk of disease in chickens [9]. Furthermore, the microbiome can also influence the quality and safety of poultry products, such as meat and eggs. For example, a disrupted microbiome in chickens can increase the risk of *Salmonella* contamination in eggs or meat [9]. Therefore, it is important for poultry producers to maintain a healthy gut microbiome in chicken by providing a balanced diet, reducing stress, and avoiding overuse of antibiotics.

Diet is one of the most influential factors shaping the gut microbiota in poultry. Dietary components that escape host digestion serve as substrates for intestinal bacteria, modulating microbial composition and function [10]. For example, wheat-based diets promote higher proportions of beneficial bacteria like *Bifidobacterium*, while corn-based diets favor *Lactobacillus* spp. [8]. Feed additives such as prebiotics and phytogenics can further enhance gut health by promoting beneficial bacterial growth and suppressing pathogens like *Clostridium perfringens* [11]. Additionally, fermentable sugars have been shown to increase microbial diversity and improve nutrient utilization [10]. These diet-induced changes in microbiota composition can influence digestion efficiency, immune modulation, energy homeostasis, and even disease susceptibility in poultry [12].

Differences in microbial communities among poultry breeds have also been observed. Breed-specific microbiomes are shaped by genetic background, environmental factors, and dietary interventions. For instance, native chicken breeds often exhibit higher microbial diversity compared to commercial broilers or layers due to differences in rearing environments and feed access [13,14,15]. Studies have shown that indigenous breeds harbor distinct microbial taxa that may confer unique health benefits or adaptations to specific environments. For example, a higher relative abundance of Firmicutes and *Lactobacillus* has been reported in native chickens from high-altitude regions compared to commercial breeds raised at lower altitudes [14]. These differences underscore the importance of considering breed-specific microbiome dynamics when designing feeding strategies.

Crickets are edible insects that are considered nutritious, protein-rich, and cost-effective [16,17]. They also contain chitin and other fibers that have recently been reported to improve gut health and reduced plasma tumor necrosis factor alpha (TNF-α) [18]. In addition to their nutritional value, crickets are also more environmentally sustainable than traditional livestock feed ingredients such as soy or fishmeal, as they require fewer resources such as land, water, and feed to produce the same amount of protein [19,20]. Nevertheless, the use of crickets as a feed ingredient for chickens is still a relatively new aspect, and more studies are needed to fully understand the potential benefits and drawbacks.

Subsequently, we hypothesized that dietary supplementation with house crickets (*Acheta domesticus*) will modulate the gut microbiota of Thai indigenous chicken breeds by enriching beneficial bacteria (e.g., *Lactobacillus, Ruminococcaceae*) and reducing pathogens while exhibiting breed-specific responses that enhance gut health and productivity. This aligns with the broader goal of identifying sustainable strategies to improve poultry health and preserve rare indigenous breeds. Therefore, this study aimed to investigate the impact of house crickets on the gut microbiota of Thai indigenous chicken [21] using 16 RNA analysis of cecum and ileum.

## 2. Materials and Methods

### 2.1. Ethical Approval

All chickens were raised in an animal unit (Registration number: B2560/00051.001) at the Faculty of Veterinary Medicine, Kasetsart University, Bangkok, Thailand following the strict animal use protocol approved by the Ethical Review Board of the Office of National Research Council of Thailand (NRCT, License No. U1-03179-2559) and the Institutional Animal Care and Use Committee of Kasetsart University, Bangkok, Thailand (ACKU65-VET-014).

### 2.2. Chicken Samples and Rearing Management

This study included three strains of indigenous chickens: the Betong Chicken (KU line), as described by Bungsrisawat et al. (2018) [21]; the white feather, black bone chicken; and the black feather, black bone chicken. Notably, Betong Chicken (KU line) is a well-characterized local breed selected for its superior meat quality and growth performance. White feather with black bone chicken is traditionally valued for its medicinal properties in Thai culture, and black feather with black bone chicken is known for its high antioxidant content in the meat and traditional medicinal relevance. Each strain was divided into two groups: a control group that was fed a standard diet without crickets, and a test group that was fed a diet supplemented with crickets (Table 1). Each dietary group was distributed across three replicate pens, with an effort to maintain equal numbers of birds per pen within each strain.

All chicken samples were housed in the Animal Experiment Room 1, Chalermphrakiat 6 Building, Faculty of Veterinary Medicine, Kasetsart University. The facility was thoroughly cleaned and disinfected to ensure a purified environment with adequate ventilation.

The chickens were kept in fourteen experimental pens, each measuring 1.2 m × 1.5 m, which is 6.9 × 6.8 square meters. Stocking density was therefore approximately 1.67 birds/m^2^ (based on 3 birds per 1.8 m^2^ pen). Each pen housed 12 chickens. The TC, TT, WC, and WT groups were allocated three replications, while BC and BT were not. The environmental conditions were carefully controlled to maintain a temperature of approximately 34 °C, a relative humidity between 50% and 70%, and a light intensity of 10 Lux for 5 days; the temperature was gradually decreased by 2 °C per week to 26 °C, which was maintained until the end of the experiment at 11 weeks. Air circulation was ensured through the use of fans for ventilation, and a light-dark cycle of 12 h of light and 12 h of darkness was maintained. Sawdust was used as bedding material in all pens with a thickness of about 5 cm depth. Each pen was equipped with a feeder and waterer to provide ad libitum access to feed and water.

Throughout the experimental period, standard management practices were followed to ensure that all chickens had access to feed and water. Vaccinations were administered according to the Department of Livestock Development’s guidelines. Specifically, the Newcastle disease and infectious bronchitis combined vaccine was given at ages 1 and 3 weeks.

### 2.3. Feed and Design

At the beginning of the experiment, one-day-old chicks were fed either control or test diets simultaneously until they reached twenty-one days of age by starter diet. After that, the finisher diet was given to each experimental group until the study was completed at 11 weeks. The diets for the control group and the test group were formulated as shown in Table 2. The diets were analyzed on a chemical composition based on the Association of Official Analytical Chemists (AOAC) [22].

### 2.4. Sample Collection

All samples were collected when the poultry reached 11 weeks of age. Four chickens were randomly collected from each diet group (*n* = 56), resulting in a total of 112 gut samples. To ensure humane handling, the chickens were suspended in an inverted position on a chain track equipped with leg restraints. They were then guided towards an electrified water bath where their heads were submerged. An alternating current of medium voltage and low frequency was applied, set at 50 V (with a current range of 48–52 mA) and a frequency of 50 Hz, for a duration of 10 s. This resulted in a rapid loss of consciousness. While the chickens were unconscious, both jugular veins were manually cut to ensure complete exsanguination. Following exsanguination, the abdomen was opened, and the gastrointestinal tract was carefully excised. The cecal and ileal contents were gently transferred into 15 mL sterile tubes for microbiota analyses, following the procedures outlined by Meijerink et al. [23]. The samples were immediately stored at −80 °C to preserve their integrity until further processing.

### 2.5. DNA Sequencing

Fecal samples from the cecum or food debris from the ileum were collected and kept at −20 °C until tested. Then, approximately 20–30 mg of the samples were used for DNA extraction using ZymoBIOMICS™ DNA Miniprep Kit (ZYMO Research, Irvine, CA, USA) following the manufacturer’s recommendation. The concentration and purity of the extracted DNA were measured by NanoPhotometer^®^ (Implen, München, Germany).

### 2.6. Amplification of the Full-Length 16S rDNA

The full-length 16S rDNA gene (1.5 kb) was amplified following the conditions and primers as described previously [PMID: 36082397]. Briefly, each PCR reaction was comprised of Phusion Plus DNA Polymerase [0.4 Units] (Thermo Scientific, Waltham, MA USA), 1X Phusion™ Plus buffer, each primer [0.2 µM], dNTPs [0.2 mM], a DNA template [1 µg], and water in a final volume of 20 µL. The PCR cycling conditions were performed as the following thermal profile: 98 °C for 30 s, 25 amplification cycles (98 °C for 10 s, 60 °C for 25 s, 72 °C for 45 s), and 72 °C for 5 min. After that, the barcodes were attached to the 16S rDNA amplicons by 5 amplification cycles (98 °C for 10 s, 60 °C for 25 s, 72 °C for 45 s) based on the PCR Barcoding Expansion 1–96 (EXP-PBC096) kit (Oxford Nanopore Technologies, Oxford, UK). Finally, the barcoded DNA libraries were purified by the QIAquick^®^ PCR Purification Kit (QIAGEN, Hilden, Germany) according to the recommended protocol.

### 2.7. Amplicon Sequencing Based on Oxford Nanopore Technology (ONT)

The concentrations of the purified libraries were measured by the Qubit 4 fluorometer with Qubit dsDNA HS Assay Kit (Thermo Scientific, Waltham, MA, USA). Then, amplicons with different barcodes were equimolarly pooled, followed by purification using 0.5X Agencourt AMPure XP beads (Beckman Coulter, Indianapolis, IN, USA). Subsequently, the pooled DNA library was subjected to end repair and adaptor ligation based on the Ligation Sequencing Kit (SQK-LSK112) (Oxford Nanopore Technologies, Oxford, UK). After that, the DNA library was sequenced based on the R10.4 flow cell of the MinION Mk1C platform (Oxford Nanopore Technologies, Oxford, UK).

### 2.8. Data Analysis

Base calling with a super-accuracy model was performed by Guppy basecaller software v6.0.7 (Oxford Nanopore Technologies, Oxford, UK) [PMID: 31234903] to generate FASTQ files followed by quality assessment with MinIONQC [PMID: 30052755] and demultiplexing and adaptor trimming using Porechop v0.2.4 [https://github.com/rrwick/Porechop (accessed on 3 December 2023)]. The NanoCLUST analysis pipeline [PMID: 33079990] was applied for clustering, polishing, and taxonomic classification of the bacteria based on the Ribosomal Database Project (RDP) database [PMID: 12520046]. The abundance and taxonomic data were converted into QIIME2 format using the QIIME2 platform [PMID: 31942082] before being imported into MicrobiomeAnalyst [PMID: 31942082] for downstream processing. Low count filtering (prevalence ≥10% of samples) and low variance filtering (IQR method) were applied to remove uninformative taxa, followed by Total Sum Scaling (TSS) normalization to correct for sequencing depth variations. The processed data were used for microbial diversity analysis, including alpha diversity (Chao1 and Shannon indices) and beta diversity (Bray–Curtis index). Principal Coordinate Analysis (PCoA) was performed to visualize beta diversity differences, and the normalized data were used for further differential abundance analysis to identify taxonomic features associated with experimental conditions.

### 2.9. Statistical Analysis

Microbial diversity and differential abundance were statistically evaluated using GraphPad Prism 7 software and MicrobiomeAnalyst [PMID: 31942082]. Alpha diversity (Chao1 and Shannon indices) was assessed using the Mann–Whitney U test for pairwise comparisons and the Kruskal–Wallis test for multiple groups. Beta diversity, calculated using the Bray–Curtis index, was visualized using PCoA, and statistical differences between groups were tested using PERMANOVA with pairwise comparisons. Differential abundance analysis was performed using linear discriminant analysis effect size (LEfSe) [PMID: 21702898] with a *p*-value threshold of <0.05 and an LDA score >3, conducted via the Galaxy web server.

## 3. Results

### 3.1. Sequencing Summary and Diversity Analysis

**Figure 1:**
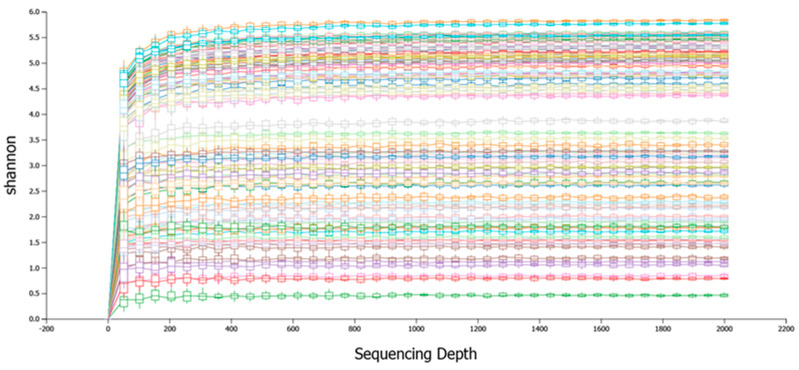
The rarefaction analysis indicates that the sequencing depth was sufficient to estimate the diversity of all the samples.

The full-length bacterial 16S rRNA gene from four groups of chickens was sequenced using high-throughput long-read nanopore sequencing. A total of 1,676,819 raw reads were obtained from 110 samples of cecum and ileum. On average, each sample had 15,244 reads. The average number of classified reads per sample was 11,756 (Table 3). To determine the overall taxonomic diversity of the microbiota present in the chicken cecum and ileum, rarefaction curves were performed to analyze the data. The analysis of both rarefaction curves demonstrated a higher rate of increase in diversity with each additional sample. It indicated that the majority of the diversity had been captured, suggesting that additional samples were unlikely to yield significantly more diversity (Figure 1).

**Figure 2:**
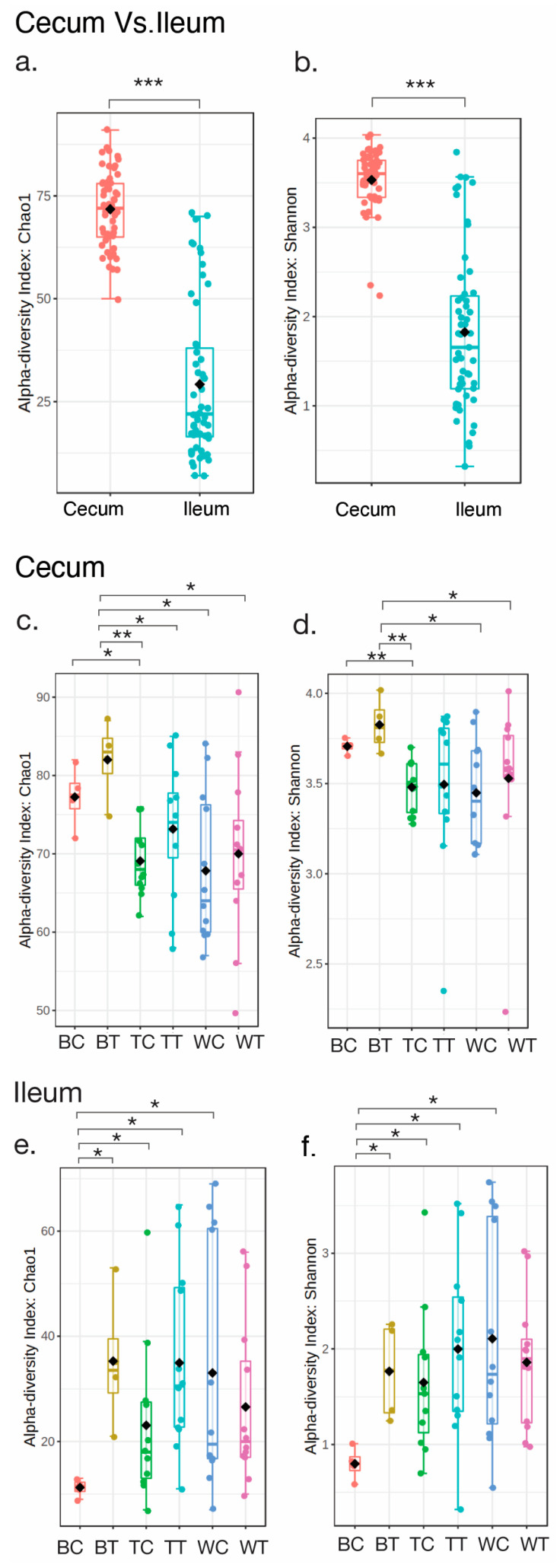
**Alpha diversity based on Chao1 index and Shannon index of different groups of chickens for cecum and ileum samples.** (**a**,**b**) show Chao1 and Shannon’s diversity indices, respectively, in comparison between cecum and ileum samples. (**c**,**d**) show Chao1 and Shannon’s diversity indices, respectively, in cecum samples. (**e**,**f**) show Chao1 and Shannon’s diversity indices, respectively, in ileum samples. “*”, “**” and “***” indicate a significant difference when compared between samples, with *p* < 0.05, *p* < 0.01, and *p* < 0.001, respectively, by Kruskal–Wallis test.

To evaluate the richness of bacterial operational taxonomic units (OTUs) in cecum and ileum, alpha diversity based on the Choa1 index and Shannon index was performed. The statistical comparisons of indices between groups were carried out with the Kruskal–Wallis test (Figure 2). As a result, there was a significantly higher level of total taxonomic richness and evenness in the cecum compared to the ileum (Figure 2a: *p* < 0.001 and Figure 2b: *p* < 0.001). Despite being fed crickets, the levels of species richness and evenness of cecum were very similar between controls and cricket-fed samples (Figure 2c,d). Nevertheless, the richness and evenness of OTUs were significantly increased in BT than in BC of ileum samples (Figure 2e,f). Furthermore, BT showed a significantly higher level of alpha diversity than other groups of chickens in the cecum (Figure 2c,d), whereas BC exhibited the lowest alpha diversity of bacterial OTUs among different groups of chickens in the ileum samples (Figure 2e,f).

**Figure 3:**
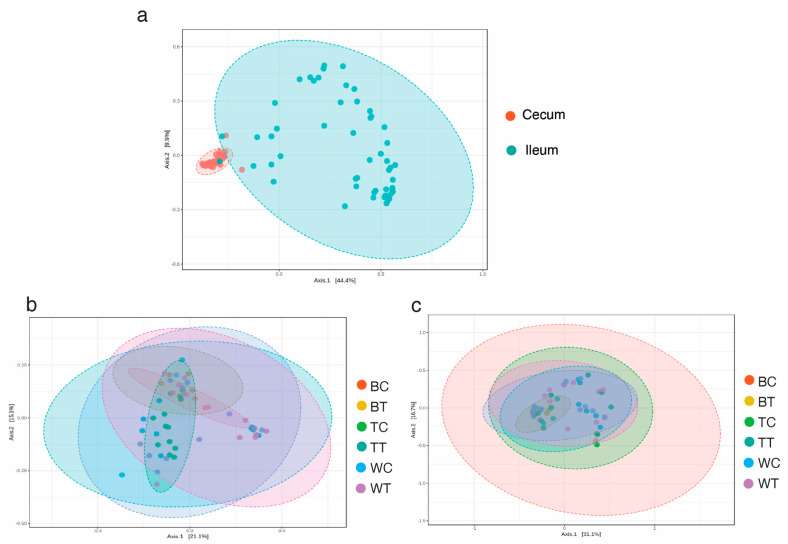
Beta diversity measured by Bray–Curtis dissimilarity. (**a**) Cecum vs. ileum, (**b**) cecum samples, and (**c**) ileum samples.

Similarly, the analysis of beta diversity based on Bray–Curtis dissimilarities (PERMANOVA *p*-value = 0.001) showed a clear clustering of cecum and ileum samples (Figure 3a). On the other hand, compositional differences were not observed among groups of chickens (Figure 3b,c). Therefore, these results suggest that the relative abundance of these OTUs was similar across the different groups of chickens.

### 3.2. Relative Abundance of Bacteria in Cecum and Ileum Samples

**Figure 4:**
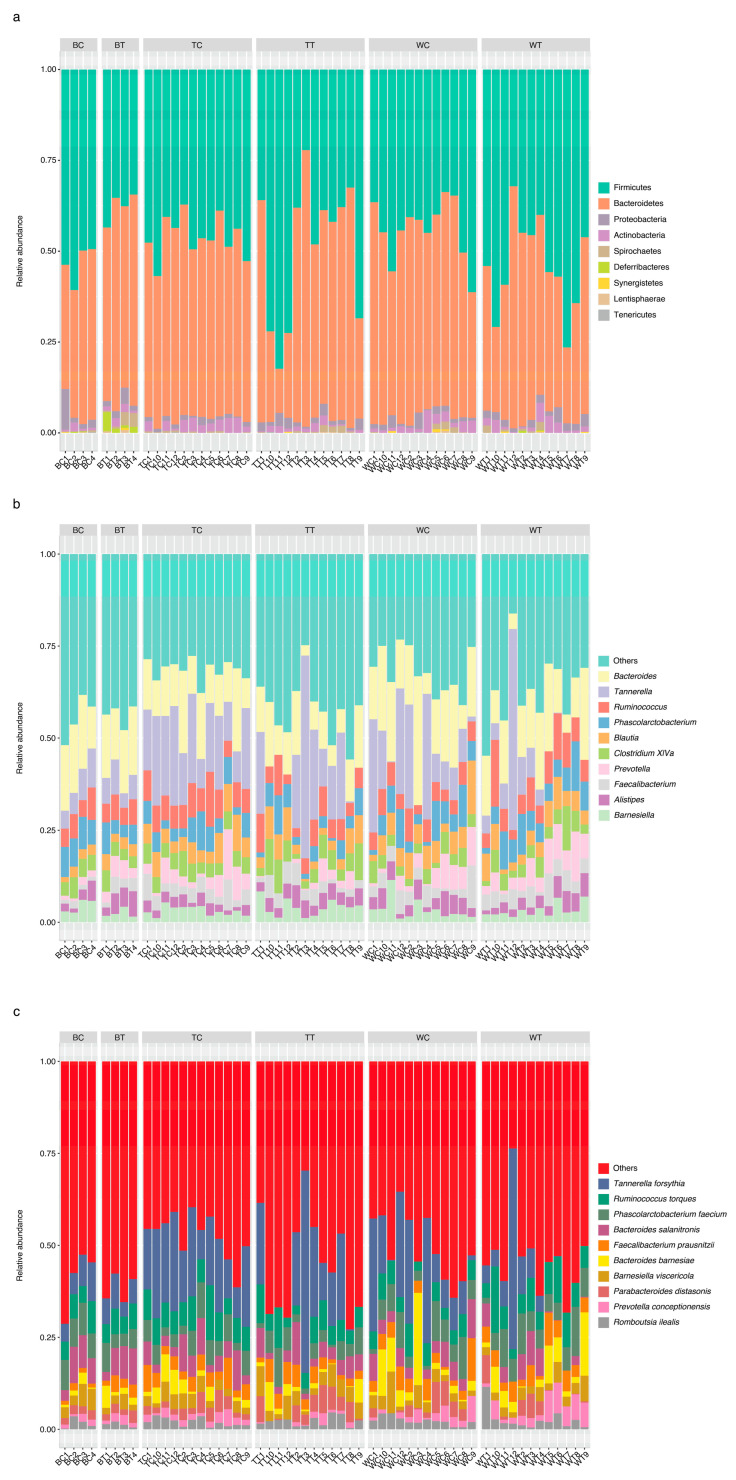
Relative abundance of bacteria in cecum samples. (**a**) Top-10 phyla, (**b**) top-10 genera, and (**c**) top-10 species level in cecum samples.

From Figure 4a, it can be observed that the microbiome in the cecal samples of the three groups of chickens was dominated by Firmicute bacteria (48.16 ± 12.37%) and Bacteroidetes (47.2 ± 12.53%), and the other phyla present were Proteobacteria (1.9 ± 1.64%) and Actinobacteria (1.84 ± 1.41%). On the other hand, Deferribacteres (2.21 ± 2.08%) was predominantly found in BT, whereas Tenericutes (0.03 ± 0.08%), Spinochaetes (0.56 ± 0.92%), and Lentisphaerae (0.05 ± 0.15%) were found in some samples across the groups. Furthermore, an overlap in the genera present in the cecum of the control group and the cricket-fed group was observed at the genus level in the microbiota. The top 10 genera were (in alphabetic order) *Alistipes*, *Bacteroides*, *Barnesiella, Blautia*, *Clostridium cluster XIva*, *Faecalibacterium*, *Phascolarctobacterium*, *Prevotella*, *Ruminococcus*, and *Tannerella* (Figure 4b). The further analysis of species levels were as follows: *Bacteroides barnesiae, Bacteroides salanitronis*, *Barnesiella viscericola*, *Faecalibacterium prausnitzii*, *Parabacteroides distasonis*, *Phascolarctobacterium faecium*, *Prevotella conceptionensis*, *Romboutsia ilealis*, *Ruminococcus torques*, and *Tannerella forsythia* (Figure 4c).

**Figure 5:**
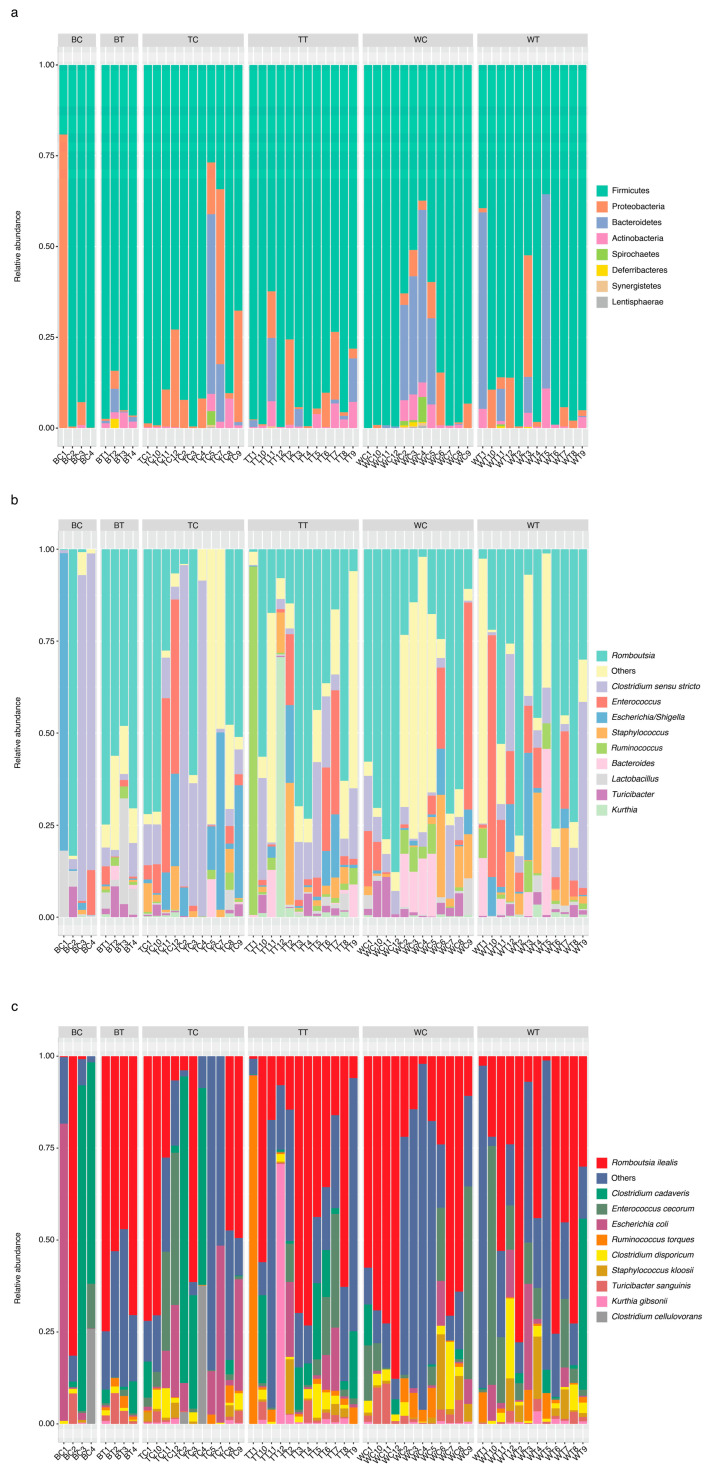
Relative abundance of bacteria in ileum samples. (**a**) Top-10 phyla in ileum, (**b**) top-10 genera in ileum, and (**c**) top-10 species level in ileum.

Similarly, Firmicute bacteria (82.9 ± 22.05%) were also presented as the major phylum across the groups of chickens in ileum samples (Figure 5a). The other phyla that were also found in both cecum and ileum included Bacteroidetes (26.98 ± 24.25%), Proteobacteria (4.92 ± 10.34%), Actinobacteria (1.91 ± 2.1%), Deferribacteres (0.14 ± 0.6%), Spinochaetes (0.4 ± 1.03%), Synergistetes (0.05 ± 0.16%), and Lentisphaerae (0.04 ± 0.12%) (Figure 4a and Figure 5a). Nevertheless, Bacteroides was one of top-ten genera found in both cecum and ileum samples (Figure 4b and Figure 5b). Other genera were present in ileum but absent in the cecum, including *Clostridium* sensu stricto, *Enterococcus, Escherichia-Shigella, Kurthia, Lactobacillus, Romboutsia, Ruminococcus 2,* and *Staphylococcus* (Figure 5b). Moreover, identification at the species level presented as follows: *Clostridium cadaveris*, *Clostridium cellulovorans*, *Clostridium disporicum*, *Enterococcus cecorum*, *Escherichia coli*, *Kurthia gibsonii*, *Romboutsia ilealis, Ruminococcus torques*, and *Staphylococcus kloosii* (Figure 5c).

### 3.3. Linear Discriminant Analysis of Effect Size (LEfSe)

**Figure 6:**
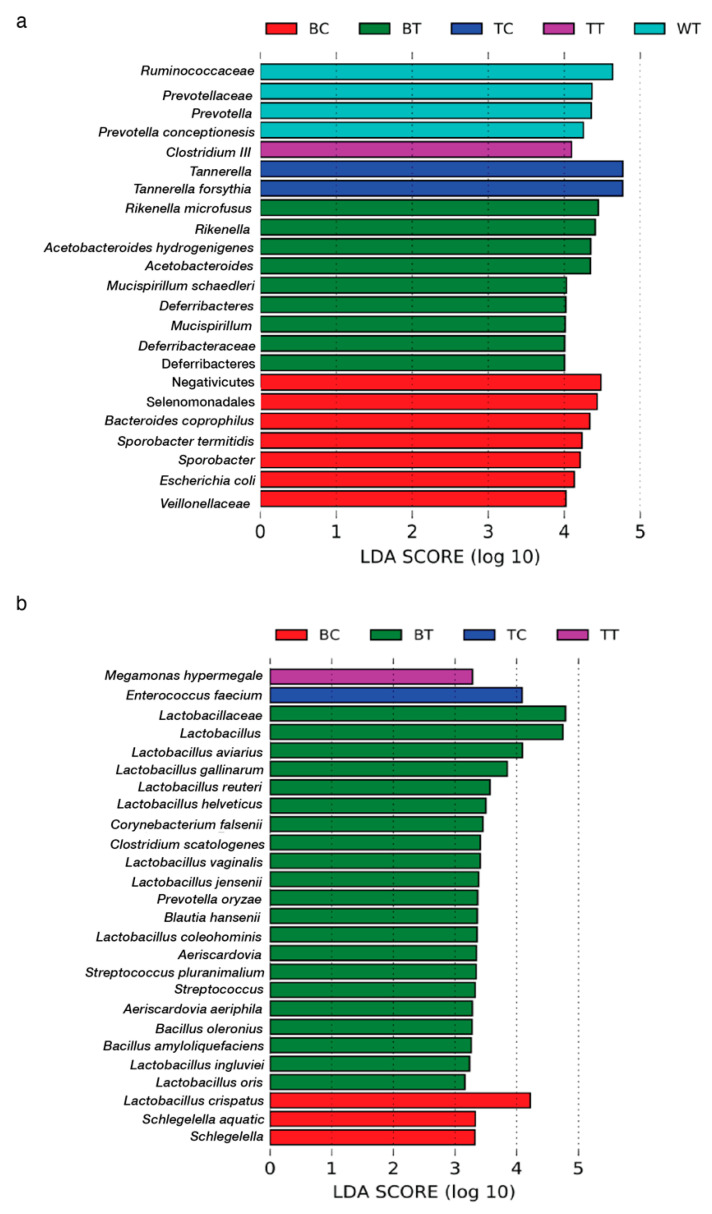
Linear discriminant analysis effect size in samples. (**a**) Cecum and (**b**) ileum.

LEfSe analysis (for *p* < 0.05, Kruskal–Wallis test) was conducted on gut microbiota samples from both the cecum and ileum of various chicken groups to obtain further insights into the differential abundance of gut bacteriomes (Figure 6). For cecum samples, *Veillonellaceae*, *Escherichia. coli*, *Sporobacter*, *Sporobacte termitidis*, *Bacteroides coprophilus*, Selenomanadales, and Negativicutes were found in BC. In contrast, *Rikenella microfusus*, Rikenella, *Acetobacteroides hydrogenigenes*, Acetobacteroides, *Mucispirillum schaedleri*, Deferribacteres, *Mucispirillum,* Deferribacteraceae, and *Deferribacteres* were differentially identified in BT. Moreover, TC cecum samples were differentiated by the presence of *Tannerella* and *Tannerella forsythia*, while *Clostridium* III was found in TT samples. For the WT cecum samples, the bacteria found in differential abundance were *Ruminococcaceae*, *Prevotellaceae*, and *Prevotella* (Figure 6a).

In contrast, Lactobacillaceae exhibited a differential abundance in the ileum of BT, including species such as Lactobacillus aviaries, Lactobacillus gallinarum, Lactobacillus reuteri, Lactobacillus vaginalis, Lactobacillus jensenii, Lactobacillus coleohominis, Lactobacillus ingluviei, and Lactobacillus oris. Additionally, Lactobacillus cripatus was also found in the ileum of BC. In the case of the ileum of BC, Schlegelella aquatic was also present (Figure 6b). Apart from Lactobacillaceae, the ileum of BT also contained Corynebacterium falsenii, Clostridium scatologenes, Prevotella oryzae, Blautia hansenii, Aeriscardovia aeriphila, Streptococcus pluranimalium, Bacillus oleronius, and Bacillus amyloliquefaciens. On the other hand, Megamonas hypermegale and Enterococcus faecium were the only species with a differential abundance found in the ileum of TT and TC, respectively (Figure 6b).

## 4. Discussion

This study provides novel insights into the microbiota of the cecum and ileum in Thai indigenous chicken breeds, emphasizing the potential benefits of feeding house crickets. Our findings align with previous research indicating that Firmicutes, Bacteroidetes, and Proteobacteria are the dominant phyla in the gastrointestinal tract of chickens (*Gallus gallus*), consistent across both cecum and ileum samples irrespective of breed (Figure 4a and Figure 5a) [24,25]. Given the distinct functional roles of different sections of the chicken’s gastrointestinal tract, it is reasonable to expect variations in ecological niches and taxonomic compositions when comparing cecum and ileum samples [26]. This study contributes to a deeper understanding of the gut microbiota in indigenous chicken breeds, which is crucial for optimizing their health and performance.

Cecum samples have been reported to exhibit higher diversity [27,28,29] due to their role as sites for storing undigested feed materials for 12–20 h, along with the presence of water, enabling the fermentation of carbohydrates present [30]. Thus, Figure 2a,b also confirmed findings from previous studies.

The gut microbiota plays a vital role in the digestion of nutrients from animal feed and extracting energy from dietary fibers. Moreover, it has been reported to promote the development of the immune system and contribute to the overall health and productivity of chicken. Through strategic feeding practices, the gut microbiota can be modulated. Therefore, it is important for farmers and poultry producers to manage the microbiome in chickens by offering a well-balanced diet and minimizing stress [31]. Insects are recognized as an excellent source of protein and are rich in various bioactive compounds, including chitin, antimicrobial agents, and lauric acid [32]. Several studies have demonstrated the potential of insects to positively influence the gut microbiota of animals, including the yellow mealworm (*Tenebrio molitor)*, housefly (*Musca domestica*), black carp (*Mylopharyngodon pieeus*), super mealworm (*Zophobas morio*), and black soldier fly (*Hermetia illucens*) [33].

For house crickets (*Acheta domesticus*), both dried and undried forms have been reported to contain numerous macro and micronutrients [29,34,35]. The dried forms, in particular, are richer in proteins and minerals [36]. The microbiological profiles of house crickets in both dried and undried forms have been reported to include aerobic mesophilic spore-forming bacteria, the *Bacillus cereus* group, Enterobacteriaceae, *Staphylococcus aureus* coagulase, yeasts, and molds. The presence of spore-forming bacteria such as *B cereus* and *S. aureus* has raised concerns about food safety [36]. Nevertheless, implementing good hygiene practices at all stages of the house cricket’s production process could contribute to improving microbiological safety.

The benefits of house cricket supplementation may be attributed to several bioactive compounds present in crickets. Crickets are rich in chitin, antimicrobial peptides, lauric acid, and other bioactive compounds that can modulate gut microbiota composition and function. Chitin, a structural polysaccharide found in insect exoskeletons, acts as a prebiotic by selectively stimulating the growth of beneficial gut bacteria such as Lactobacillus and Bifidobacterium [37]. Its degradation by gut microbes produces chitooligosaccharides, which exhibit anti-inflammatory properties and promote gut barrier integrity. Antimicrobial peptides present in crickets may suppress pathogenic bacteria while promoting beneficial microbial populations, potentially explaining the observed increase in taxa like *Rikenella* and *Lactobacillus,* both known for reducing inflammation and enhancing nutrient absorption [38]. Lauric acid, a medium-chain fatty acid in crickets, has antimicrobial properties against *Gram*-positive bacteria such as *Clostridium* perfringens, contributing to a healthier microbial balance by reducing harmful bacteria while allowing beneficial taxa like *Ruminococcaceae* to thrive [39]. Additionally, the fermentation of cricket-derived fibers by gut microbes likely enhances short-chain fatty acid (SCFA) production (e.g., butyrate), which is critical for maintaining gut health by providing energy to colonocytes, reducing inflammation, and strengthening the intestinal barrier [40]. These mechanisms collectively support the hypothesis that house cricket supplementation can positively influence gut microbiota composition and function, thereby improving health outcomes such as nutrient absorption, immune modulation, and growth performance.

The results from the LEfSe analysis (Figure 6) revealed that each chicken breed has a distinct differential abundance of gut bacteriomes. Thus, the result confirmed that taxonomic differences in the gut microbiome can be found among different chicken breeds [13]. The observed differential abundance of beneficial bacteria among breeds highlights the unique microbiome dynamics influenced by diet and genetic factors.

The increased abundance of *Rikenella* in BT chickens is particularly noteworthy due to its reported role in alleviating intestinal inflammation [41]. This suggests that feeding house crickets may enhance gut health in BT chickens by reducing inflammation, potentially improving nutrient absorption and overall resilience to gastrointestinal disorders. Similarly, the presence of *Deferribacteraceae*, known for its role in upregulating amino acid and vitamin metabolism [42], suggests enhanced metabolic efficiency in BT chickens. These metabolic improvements could translate into better feed conversion ratios and improved growth performance, which are critical for optimizing poultry production.

The differential abundance of *Ruminococcaceae* in WT chickens further underscores the potential for cricket-based diets to modulate gut microbiota. *Ruminococcaceae* is well-documented for its ability to produce butyrate and other short-chain fatty acids (SCFAs), which are essential for maintaining gut integrity and promoting anti-inflammatory effects [43]. While this benefit was observed in WT chickens, its absence in BT chickens suggests breed-specific microbiome responses to dietary interventions.

The increased abundance of *Lactobacillus* in the ileum of BT chickens after being fed house crickets is particularly significant. As a well-known probiotic, *Lactobacillus* has been extensively reported to improve intestinal health, enhance growth performance, and strengthen immune responses in poultry [44]. This finding highlights the potential for cricket-based diets to selectively enrich beneficial microbial populations, particularly in rare breeds like BT chickens. Given that BT chickens are characterized by slow growth rates and low chick numbers, these microbiome enhancements could provide a pathway to improve their productivity while preserving their unique genetic traits.

Furthermore, black meat chickens such as BT, Kadaknath from India, and black Silkie from China are valued for their rich content of bioactive compounds like carnosine and anserine, which have antioxidant and anti-inflammatory properties [45]. The observed microbiome shifts suggest that cricket-based diets may complement these inherent qualities by further enhancing gut health and nutrient metabolism. This could increase their market value and commercial viability while maintaining their cultural significance.

Despite these promising findings, it is important to acknowledge the limitations of this study. The small sample size for BT chickens may limit the generalizability of our results. Future studies should include larger sample sizes to validate these findings. Additionally, while this study focused on bacterial 16S rRNA profiling, future investigations should explore archaeal communities using archaeal-specific primers or shotgun metagenomics to assess methane-producing potential and other functional aspects of the gut microbiome.

## 5. Conclusions

This study demonstrates that feeding house crickets can positively influence the gut microbiota of Thai indigenous chicken breeds, particularly BT chickens. The enrichment of beneficial bacteria such as *Rikenella, Deferribacteraceae, Ruminococcaceae*, and *Lactobacillus* underscores the potential of cricket-based diets to enhance gut health, nutrient metabolism, and overall productivity. These findings provide a foundation for developing sustainable feeding strategies that optimize poultry health while preserving rare indigenous breeds.

## Figures and Tables

**Table 1:** Chicken samples used in this study.

Group	Description	Code	Number
1	Betong Chicken (KU line) control	TC	11
	Betong Chicken (KU line) test fed on crickets	TT	12
2	White feather, black bone control	WC	12
	White feather, black bone test fed on crickets	WT	12
3	Black feather, black bone control	BC	4
	Black feather, black bone test fed on crickets	BT	4

**Table 2:** Ingredients and chemical composition of control and test diets in different periods.

Items	Starter Diet		Finisher Diet	
	Control Group	Test Group	Control Group	Test Group
**Ingredients (% Fresh Matter)**				
Maize	62.05	64.35	60.04	66.11
Defatted soybean meal	30.7	17.22	27.618	13.2
Cricket powder	0	15	0	15
Palm oil	3.92	0	5.27	0
Cellulose	0	0	3.8	2.1
Calcium carbonate	1.33	1.35	1.53	1.59
Dicalcium phosphate	0.71	0.75	0.29	0.29
Vitamin and mineral premix ^1^	0.25	0.25	0.25	0.25
L-Lysine	0.25	0.29	0.24	0.31
Salt	0.24	0.24	0.24	0.24
DL-Methionine	0.21	0.2	0.332	0.5
Sodium bicarbonate	0.20	0.20	0.20	0.20
L-Threonine	0.07	0.08	0.12	0.14
Choline chloride	0.07	0.07	0.07	0.07
**Calculated chemical composition (% fresh matter)**				
Metabolizable energy (kcal/kg)	3112	3112	3100	3100
Crude protein	19.5	19.5	18.0	18.0
Ether extract	6.51	6.23	7.73	6.23
Crude fiber	3.18	4.74	6.59	6.61
Calcium	0.90	0.90	0.80	0.80
Available phosphorus	0.45	0.45	0.30	0.30
Lysine	1.20	1.19	1.17	1.17
Methionine	0.51	0.50	0.61	0.77
Methionine + Cysteine	0.84	0.89	0.91	0.91
Threonine	0.80	0.80	0.79	0.79
**Analyzed chemical composition (% fresh matter)**				
Moisture	10.1	10.4	10.4	10.2
Crude protein	19.8	19.7	18.3	18.4
Ether extract	6.52	6.53	7.97	7.05
Crude ash	5.11	5.15	5.29	5.22
Crude fiber	3.23	4.84	6.21	6.23

^1^ Vitamin and mineral premix (Feed Specialties Co., Ltd.; Pathumthani, Thailand) were supplied per kilogram of diets at 2,500,000 IU of vitamin A; 1,000,000 IU of vitamin D3; 7000 IU of vitamin E; 700 mg of vitamin K; 400 mg of vitamin B1; 800 mg of vitamin B2; 400 mg of vitamin B6; 4 mg of vitamin B12; 30 mg of biotin; 3111 mg of Ca pantothenate acid; 100 mg of folic acid; 15,000 mg of vitamin C; 5600 mg of vitamin B3; 10,500 mg of Zn; 10,920 mg of Fe; 9960 mg of Mn; 3850 mg of Cu; 137 mg of I; and 70 mg of Se.

**Table 3:** An overview of the sequencing and reads classification details (average ± standard deviation) for each group of chickens and feeds.

Strains of Chicken	Feed Group	Sample Type	*n*	Total Raw Reads	Total Retained Reads
Betong Chicken (T)	Control (C)	Cecum	11	15,787 ± 1603	12,736 ± 1384
		Ileum	11	18,090 ± 10,472	12,401 ± 6126
	Fed on crickets (T)	Cecum	12	15,907 ± 1496	13,591 ± 1407
		Ileum	12	13,406 ± 1783	10,610 ± 2190
White feather (W), black bone	Control (C)	Cecum	12	16,516 ± 2594	13,185 ± 2048
		Ileum	12	13,016 ± 3154	9063 ± 2544
	Fed on crickets (T)	Cecum	12	15,287 ± 1861	12,485 ± 1494
		Ileum	12	12,149 ± 1875	8415 ± 1802
Black feather (B), black bone	Control (C)	Cecum	4	16,621 ± 2405	13,469 ± 2052
		Ileum	4	20,280 ± 11,772	14,699 ± 7338
	Fed on crickets (T)	Cecum	4	16,658 ± 884	13,118 ± 620
		Ileum	4	13,644 ± 1004	10,837 ± 1206

## Data Availability

The sequencing datasets used in this study are publicly available in the NCBI Sequence Read Archive (SRA), BioProject ID: PRJNA1054336.

## Abbreviations

The following abbreviations are used in this manuscript:

TC	Betong Chicken (KU line) control
TT	Betong Chicken (KU line) test fed on crickets
WC	White feather, black bone control
WT	White feather, black bone test fed on crickets
BC	Black feather, black bone control
BT	Black feather, black bone test fed on crickets
